# Anomalous and Fatal Case of Disease

**Published:** 1833

**Authors:** A. W. Bowen


### Art. II.—Anomalous and Fatal Case of Disease.

*Russia, Herkimer Co. N. Y. 10th August, 1833.*

*To the Editor of the Western Journal of the Medical and Physical Sciences:*

*Sir:—I send you the following case for publication, if you think it worthy, in hopes you may add some remarks calculated to solve the problem—what was the cause of death in this instance?*

*I am, Sir, very respectfully,*
*Your obedient servant,*
*A. W. BOWEN.*

Charles Dyer, aged 15, rather plethoric in habit, and of fine, healthy constitution, while binding a sheaf of grain in the field, early in August, 1832, was suddenly thrown to the ground by the breaking of the band. He fell on his face, and was considerably stunned and bruised. He was bled soon afterwards, and in a day or two recovered so well as to go about his ordinary labor. On the first of September following, while hard at work, pitching sheaves, he felt something suddenly give way at the pit of his stomach, immediately became faint, and was scarcely able to reach the house, though near by. I saw him about dusk. He was unable to sit up a minute; had much pain and soreness in the epigastrium; pulse small, eighty in a minute, fever. I took from the arm about ten ounces of blood, and while it was flowing, the pulse was reduced to less than fifty in a minute. Indeed it was the reduction of the pulse, rather than any relief afforded, that induced me to untie the ligature. Left him a cathartic. Visited him next morning. Cathartic had not operated,—could not sit up at all, soreness at the pit of the stomach increased, pulse 75. At this time I perceived distinctly, what I felt confusedly, the day before, viz. a regular pulsation extending from the end of the sternum to an inch below the navel. Repeated cathartic (jal. and cal.) and left an epispastic, to be applied long enough to irritate the surface. I saw him the third day. His bowels had been moved freely—

his fever was gone, and the epispastic had relieved the soreness, which appeared to be in the muscles. But the pulsation at the epigastrium was now more distinct, both to the eye and touch, and its lateral extent could be more accurately determined, being nearly two inches. His pulse was almost natural. On the fourth day, after the accident, there was a considerable return of the vascular excitement, which was relieved by venesection to eight ounces, and a cathartic. At this time bleeding produced no other effect on the pulse than slightly to relieve its force. From this day forward, for many weeks, there was but little variation in his symptoms. He was annoyed with a dull pain in the epigastric region, which kept him continually moaning while awake, but while asleep left him. His rest at night was pretty good, and an appetite not altogether wanting. His tongue was red—after the first day, considerably so—resembling a piece of beef steeped in brine of saltpetre; his bowels continued torpid—requiring large quantities of physic to move them. For this purpose he used sulphate magnesia at first—then substituted rhubarb, and afterwards a pill aloes, rhubarb, cinnamon and colombo. His voice became nearly inaudible, and at the end of two months he could not be heard, unless the ear was put close to his mouth. His muscular energies were prostrated and he became perfectly helpless, his extremities, particularly his feet and legs, were constantly cold, and became very much emaciated, and so weak that he could not raise his thighs. At the end of six months, he could not raise or even hold up his head, or help himself in any other way than to use a spoon with the hand, while his arm was resting on the elbow,—a power that was reserved to him during life. Occasionally when obliged to take large quantities of cathartic medicines, his stomach would throw them off, this would not last longer than a few hours after which he would feel somewhat relieved. He generally felt best after having evacuations from the bowels. In the spring following the accident, he had soreness of the throat, attended with a cough, bloody sputu and a flushed cheek; but these symptoms lasted only a few days. When medicines disagreed with him, they occasioned a *distress* as he termed it—not pain nor fever, but an uneasy feeling at the stomach. The blue pill invariably gave him this sensation. There was no disturbance of the brain apparent;—all the faculties of his mind were preserved to the last, in their usual activity; and he presented to a spectator, the appearance of a person of fine healthy and rather athletic constitution, suddenly deprived of his corporeal powers and inevitably wasting away under the enervating effects of his confinement. During the last three months of his existence, the only wonder was that life should be so tenacious of the worn-out body. From the first, the only traces of disease apparent, were the redness of his tongue, costiveness, distress and pulsation at the epigastrium—to which point, the patient referred all his morbid sensations.

Although the pulsation was visible and remarkably distinct, still there was no external tumor; nor was there intermission or other irregularity of the pulse; but upon examination with the fingers we should say, if such a thing were possible, it had the feel of an enlargement of the abdominal aorta, from the diaphragm quite down to its bifurcation. Aware that the ordinary pulsation of the aorta may be felt and in some instances seen, when the muscles are relaxed—still the hardness of a tumor—its well-defined limits—the soreness upon pressure are wanting in a healthy person to complete the description of the present case. Nor could we otherwise account for the failure of his voice—coldness, loss of strength, emaciation of his lower extremities, torpidness of the bowels, and finally, the general decay of the system, which commenced immediately after his confinement. Continually wasting away as he inevitably was, still life clung to his emaciated body until the 31st July, 1833, almost a year from the time he received the injury, and more than eleven months after his confinement began. His death was preceded during the last two days by vomiting and purging; the former, continuing as long as his strength permitted the effort.

He was visited by the neighboring physicians, several of whom are men of experience and eminence in the profession.

Those who ventured an opinion upon the case, considered it aneurism of the aorta—others more cautious said if it were not aneurism they could not name it; while others again declared that it could not be aneurism—although as to its real nature they professed themselves equally ignorant.

Under the hope that the last opinion was correct, various remedies were proposed in the early stages of his complaint, chiefly directed towards a change of habit—such as rocking, swinging, trying to sit up; alteratives to the stomach and bowels, such as the blue pill, iodine, &c.; blisters along the spine, colchicum, and various kinds of tonics—all or any of which, invariably added to the distress at his stomach. The lightest kinds of vegetable food agreed with him best, and he always felt better after evacuations, and without any other medicines than anodynes. After a few months these became indispensable to his comfort. At first he used pulv. Dov. afterwards tinct. opii, and, finally, what agreed much better with him, a syrup of sulphate of morphia.

*Post Mortem Examination.* Fifteen hours after death with the assistance of my friends, Drs. Sears, Booth and Willoughby, I made an examination of the body. On laying the abdomen open, by an incision from the sternum to the pubis, and a lateral one through hypochondria; the first thing that attracted our attention, was a considerable distention of the stomach, which in this emaciated frame, appeared very much enlarged. Removing this, we sought at once for the aorta. This, to the surprise of most of us, was found of its natural, or perhaps rather of a diminutive size, and *perfectly sound*, quite down to its bifurcation. The remaining abdominal viscera appearing equally healthy, we next turned our attention to the chest. Here we found nothing more satisfactory. In the pericardium there was perhaps half a wine glass full of a limpid fluid, and the heart and large arteries and the lungs perfectly sound, as were, also, the liver, spleen, pancreas, intestines, mesentery, kidneys and bladder; among the whole not a trace of disease of any kind was visible, nor was there the least lesion of any organ discoverable, any where within the scope of what we intended for and believe was, a faithful examination. On opening the stomach which we had detached with its contents, we found it filled with a stercoracious fluid which had evidently been thrown in from the intestines. There were on the inner coat of the stomach, one or two small red patches of a kind of efflorescence or redness, which was so fine and in fact so indistinct, that none of us were enabled to determine whether it was a morbid appearance, or a *post mortem* effect; and, if the former, we concluded that the symptoms of cholera during the two preceding days, were more than sufficient to account for it.

Thus left in the most profound ignorance of the cause of death in this case, we have drawn up this statement in order to solicit from our professional brethren, any observations calculated to throw light upon the subject.

### Editorial Remark.

We regret that the medical gentlemen, concerned in this interesting case, did not extend their examination to the brain and spinal marrow, particularly the latter. From the symptoms and the remote cause, we suspect they would have found the spine the seat of inflammation—leading, perhaps, to effusion.

